# Efficacy of electroacupuncture on acute abdomen emergency care: study protocol for a randomized controlled trial

**DOI:** 10.1186/s13063-020-4071-3

**Published:** 2020-02-24

**Authors:** Yuan Ya Chang, Chih Wen Chiu, Chia Yun Chen, Chin Fu Chang, Tsung Chieh Lee, Lun Chien Lo, Chia Ying Lee, Kai Chang, Po Wei Chen, Chang Ju Hsieh, Yu Jun Chang, Sung Yen Huang

**Affiliations:** 1 Department of Chinese Medicine, Changhua Christian Hospital, 2F., No. 229, Xuguang Rd., Changhua City, Changhua County 500 Taiwan (Republic of China); 2 Department of Emergency Medicine, Changhua Christian Hospital, No. 135, Nanxiao St., Changhua City, Changhua County 500 Taiwan (Republic of China); 3 Graduate Institute of Statistical and Informational Science, National Changhua University of Education, No. 1, Jinde Rd., Changhua City, Changhua County 500 Taiwan (Republic of China); 4 Epidemiology and Biostatistics Center, Changhua Christian Hospital, 3F., No. 20, Jianbao St., Changhua City, Changhua County 500 Taiwan (Republic of China)

**Keywords:** Electroacupuncture, Emergency department, Abdominal pain, Ileus

## Abstract

**Background:**

Acute abdomen is a common disease in the emergency department (ED) and usually results in huge medical expenditure. To relieve abdominal pain effectively and reduce bed occupancy rate in emergency rooms, electroacupuncture is a practical method in the treatment of abdominal pain.

**Methods/design:**

Five hundred patients will be randomly and evenly divided into experimental and control groups. Both groups should have their basic information taken and their bilateral acupuncture points ( Hegu (LI 4), Neiguan (PC6), Zusanli (ST 36), Shangjuxu (ST37), Xiajuxu (ST39), Taichong (LR3), and Taibai (SP3)) will be intervened by electroacupuncture or vaccaria Seeds, in this clinical study. Electroacupuncture has been introduced to this experiment as an auxiliary technique. The experimental group will receive real electroacupuncture, but the control group will receive a placebo electroacupuncture in which transcutaneous electrical nerve stimulation will not be turned on. After the intervention, we will evaluate the difference in abdominal pain, the length of stay at the emergent observation ward, and the proportion of revisits with abdominal pain.

**Discussion:**

In Taiwan, medical expenditure is increasing annually because of the higher bed occupancy caused by acute abdominal pain in the hospital. We expect that the combined treatment of electroacupuncture and modern medical treatment will not only reduce bed occupancy and the length of ED stay but also effectively decrease the rate of readmission and revisits by 72 h. By means of electroacupuncture, the spiraling cost of health care can eventually be reduced.

**Trial registration:**

ClinicalTrials.gov identifier: NCT03199495. Registered on 27 June 2017.

## Background

Acute abdominal pain is one of the common symptoms in the emergency department (ED). In the USA, about 5 million patients have suffered from abdominal pain and visited the ED. These accounted for around 5–10% of all emergency patients [1, 2]. In 2014, Murata *et al*. demonstrated that ileus is the main factor leading to abdominal pain [3]. Around 8% of female and 9.1% of male patients had acute abdomen pain due to ileus. For patients over 60 years old, ileus is the major factor resulting in abdominal pain regardless of gender [3]. Therefore, finding out how to reduce acute abdominal pain effectively is an important task in EDs.

The definition of ileus is that partial or complete intestine is obstructed. Hence, the intraluminal contents cannot be excreted [4, 5]. Symptoms associated with ileus include (1) abdominal pain, (2) vomiting, (3) abdominal distension, and (4) constipation [6, 7]. Eighteen to forty-two percent of emergency patients need hospitalization [8]. Even though the patients do not meet admission criteria in spite of the persistent pain, basic medical cares in the emergency observation rooms are required. As a result, high levels of bed occupancy have been increasing. The longer patients stay in the emergency observation room, the more medical resources are wasted. Thus, hospital beds in emergency rooms cannot be used efficiently [9–12]. Furthermore, unscheduled return visits are the popular chief complaints; 19.9% of the patients with abdominal pain stayed in the ED over 6 h and then returned to the ED for a further stay [13, 14]. In 2010, Yeh *et al*. found that the cost of medical care for the returning ED visits is higher than for the initial ED visits and that the cost of medical care for other hospital ED visits are higher than for the original ED visit [15].

Compared with Western medicine, traditional Chinese medicine is considered a complementary medicine. Acupuncture is a safe therapy with low risk of adverse side effects in clinical practice [16]. It has been widely applied to treat gastrointestinal disease for many years. In 1996, the World Health Organization reported that 64 symptoms of gastrointestinal disease, such as epigastric pain, paralysis ileus, indigestion, and constipation, could be treated by acupuncture [17].

Electroacupuncture therapy is an auxiliary technique for acupuncture. Stimulating acupuncture points with pulse current through an electroacupuncture apparatus can increase gastric emptying and reduce the period of ileus after abdominal or laparoscopic surgery. Furthermore, side effects after the operation were reduced. For example, functional constipation could be alleviated efficiently [18, 19].

To the best of our knowledge, electroacupuncture has not been applied to the treatment of acute abdominal pain in EDs. In this study, we will collaborate with an ED in order to investigate whether electroacupuncture can alleviate acute abdominal pain effectively and reduce the length of stay in the emergency observation area. We expect that the combination of traditional Chinese medicine and Western medication can provide efficient treatment for patients and save medical resources.

## Methods/design

### Trial design

**Fig. 1:**
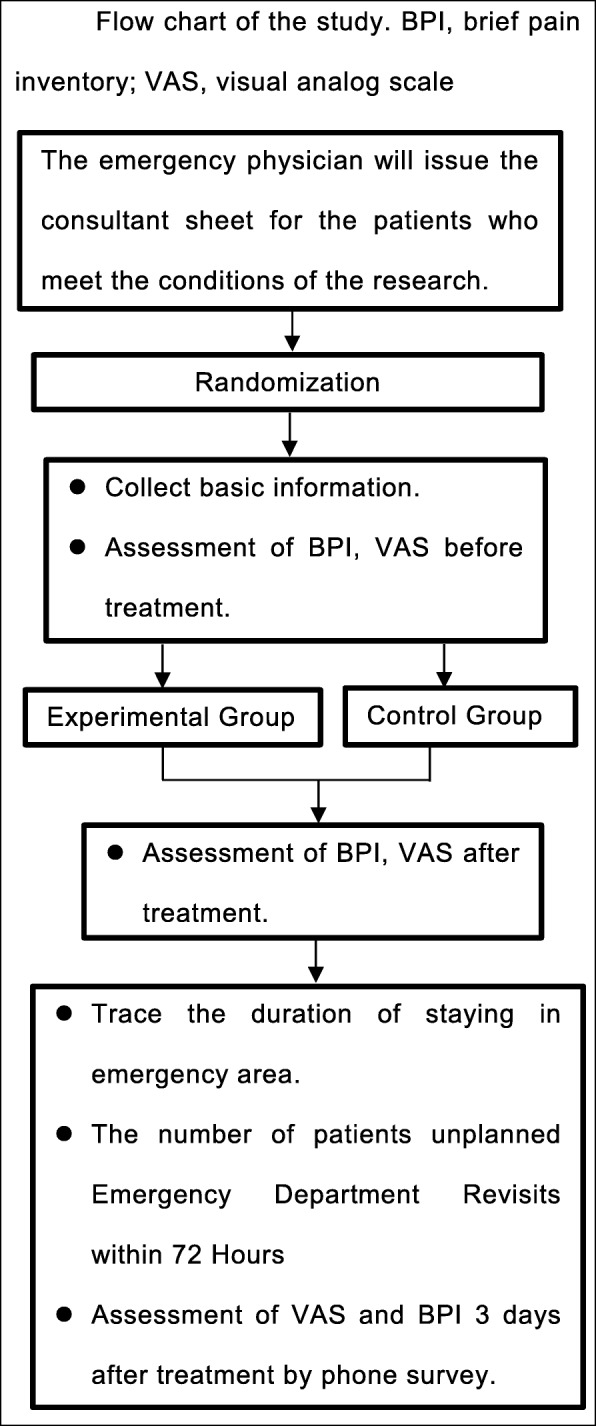
Flow chart of the study. *Abbreviations*: *BPI* brief pain inventory, *VAS* visual analog scale

This is a prospective randomized controlled clinical trial with a two-arm, parallel-group design (Fig. 1). Data analysis will be performed in accordance with the per-protocol analysis.

### Participant eligibility

We expect to recruit 500 patients and allocate them randomly to an experiment group or a control group. Patients who come to the Changhua Christian Hospital ED with acute abdominal pain will be evaluated by the attending physicians. The patients are in urgent conditions such as unstable vital signs and must stay in the hospital for further examination will be excluded by ED physicians. The consultation sheet will be issued after seeking the patients’ agreement.

#### Inclusion criteria

Participants meeting all of the following criteria are included:

1. age of 20 to 90 years, either gender;
2. the chief complaint consists of gastrointestinal symptoms, such as nausea, vomiting, diarrhea, bloating, and constipation;
3. diagnosis with International Classification of Diseases 10th revision (ICD-10) code K00-K94: Diseases of the digestive system.

#### Exclusion criteria

Participants meeting one or more of the following criteria are excluded:

1. patients are not likely to follow the instructions of the trial or cannot communicate reliably with the investigator;
2. cardiac diseases, such as arrhythmia, or equipped with cardiac pacemaker, deep brain stimulator, or other electronic implants;
3. abnormal vital signs;
4. serious injuries;
5. physical symptoms of a neurological problem;
6. the situation of ileus needs surgery;
7. pregnancy status;
8. mental disabilities or unconsciousness.

#### Participating center/physicians

This study will be conducted in the ED at Changhua Christian Hospital. All of the traditional Chinese medicine doctors in this research have more than 10 years of acupuncture experience.

#### Sample size and randomization

The sample size calculation was based on the results of our previous pilot study by using the statistical software G*Power 3.1.9.2. The minimum sample size for estimating the difference in the mean change of visual analog scale (VAS) at 15 min between the experimental and control groups with an effect size of 0.275 (means of 1.1 and 0.6, standard deviations of 2.4 and 0.9, *n* = 26 and 28, respectively) was calculated. With these parameters, a sample size of 208 subjects in each group was needed to have 80% power and 5% level of significance by independent *t* test. With a predicted attrition rate of 10%, the minimum number of subjects required at baseline was calculated to be 229 per group. Patients meet the inclusion criteria will be randomly assigned by computer in a 1:1 ratio, and the results of this random assignment will be sequentially numbered into each sealed opaque envelope containing a card labelled ‘electroacupuncture’ (experimental group) or ‘placebo electroacupuncture’ (control group). Whenever a patient participates in the study, a sealed opaque envelope will be opened by a doctor.

#### Interventions

The emergency physicians will issue the consultant sheet. After receiving the completed informed consent forms, the research assistant will interview the patients and their families, explain the plan and purpose of research, and collect basic information, including age, gender, height, weight, family medical history, history of drinking alcohol and smoking, body mass index, vital signs, blood/biochemical test, and medical imaging. The research assistant will then inform the traditional Chinese medicine physician who will give immediately medical treatment. Before treatment, the research assistant will assess the brief pain inventory (BPI) and VAS of the patients.

##### Experimental group: electroacupuncture

Patients in the experimental group will receive electroacupuncture with electrostimulation by using a Ching Ming electroacupuncture device. Disposable, sterilized acupuncture needles (length of 40 mm, diameter of 0.27 mm; Tennyson Medical Instrument Developing Co., Ltd., Taiwan) will be inserted to a depth of 15–35 mm. Needles are inserted correctly and are manually stimulated until the “De Qi” sensation is elicited. Among the seven acupunctural points mentioned above, only Hegu (LI 4), Neiguan (PC6), Zusanli (ST 36), and Taichong (LR3) with electrical stimulation are conducted. The frequency of electrical stimulation is 2 Hz, and the intensities of the stimulation are below 9.8 mA for 15 min [20]. Once it is completed, the investigators will remove the acupuncture needles and start the BPI and VAS after 15 min.

##### Control group: sham electroacupuncture

Sham electroacupuncture is delivered by Vaccaria seeds which are categorized as spherical, smooth, and hard. Patients in the sham acupuncture group receive sham electroacupuncture by using Vaccaria seeds which are secured onto the same acupuncture points as the experimental group and then covered by patches of transcutaneous electrical nerve stimulation with the power off. The control variable in this study is carried out with the electrical stimulation off. After 15 min, the investigators will remove Vaccaria seeds and patches of transcutaneous electrical nerve stimulation.

During the study, all participants will receive Western conventional therapy simultaneously, and the researchers are responsible for recording all of the data and for the safety of patients. If any hemorrhages, bruises, or swelling occur after pulling out needles, the doctor will directly make provisions for the patients. For example, putting ice packs on the bruised area can be useful for absorbing bruises. After treatment, all of the patients should be followed up by a phone interview within 3 days.

### Outcomes

#### Primary outcome

Abdominal pain is registered and evaluated by the BPI and VAS. The BPI is used to assess the severity of pain and the impact of pain on daily functions. The VAS is graded from 0 (no pain) to 10 (severe pain). Patients are evaluated at three time points in this study: before intervention (VAS-1), after intervention (VAS-2), and 3 days after the intervention (VAS-3).

#### Secondary outcome

The secondary outcome measurements are the following:

1. The duration of staying in ED after electroacupuncture treatment
2. The number of unplanned ED revisits within 72 hours.

**Fig. 2:**
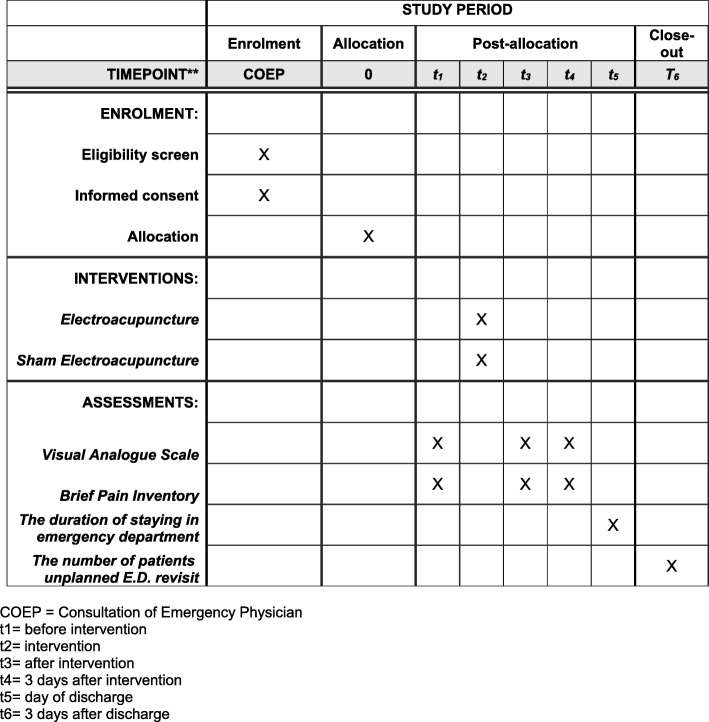
Schedule of enrollment, interventions, and assessments

The research assistant will keep tracking the direction (conditions) of post-treatment participants. Any personal information will remain confidential in a safe. Only the principal investigators can get the assessment (Fig. 2).

### Statistics

All data will be analyzed by using IBM SPSS Statistics for Windows, version 22.0 (IBM Corp., Armonk, NY, USA). Continuous variables between the two groups are compared by using the Student’s *t* test, and categorical variables between the two groups are compared by using the chi-squared test or Fisher’s exact test. A paired *t* test is used to determine whether the mean difference between post-treatment and pre-treatment VAS and BPI levels is zero. To estimate the effect of electroacupuncture treatment and adjust for the possible inter-correlations of data collected from the same patient and other covariates, we will use a generalized estimating equation to fit a linear regression model that yields robust standard errors estimated in the model.

### Data management

Each group of patients will be randomly assigned by computer in a 1:1 ratio. The medical information will be documented and stored separately from personally identifiable data. Independent personnel will record data on the paper version of a case report form and input the data to the computer database. The medical information and the group allocation will not be visible to third parties. All data will be stored in the database and inspected by a person who is not involved in this study. The doctors and statistician will not have access to these data until patient evaluations are completed. If subjects withdraw from the study, no further data will be collected from them. Data already obtained will be used only in analysis of this study. Once we complete the study, we will delete the data from the database.

## Discussion

In Taiwan, medical expenses caused by return visits have been increasing gradually year by year [15, 21–23]. In 2013, the total emergency medical expense was 20 billion New Taiwan dollars (NTDs), of which acute abdominal pain occupied 0.52%, which equals 100 million NTDs [21, 24].

Acute abdomen pain is one of the most frequent causes of admission to EDs. In the past, in Changhua Christian Hospital, we observed that most patients with acute abdomen pain were between about 20 and 90 years of age. Therefore, we will include patients up to the age of 90 years.

The BPI and VAS are tools of pain assessment. Because the BPI is indirectly derived from the scores of the questionnaire items, we use the VAS to estimate the minimum sample size for this study.

According to past studies [18, 19], the intervention of traditional Chinese medicine is a practical approach to shorten the period of acute abdominal pain. Consequently, it is possible to save medical expenditures. The aforementioned experimental procedures could effectively relieve the symptoms caused by ileus. There are seven acupuncture points: Zusanli (ST 36), Hegu (LI 4), Shangjuxu (ST37), Xiajuxu (ST39), Neiguan (PC6), Taibai (SP3), and Taichong (LR3). These points are chosen according to the literature and our clinical research [18, 19]. The fundamental combination of Neiguan (PC6), Zusanli (ST 36), Shangjuxu (ST37), and Xiajuxu (ST39) could stimulate peristalsis, increasing the gastric emptying, regulating Qi, and relieving vomiting and abdominal distention. Further collaboration with Taibai (SP3) can enhance the digestive function and avoid ileus relapse. The conjunction of Hegu (LI 4) and Taichong (LR3) could adjust metabolism and relieve tension caused by acute abdominal pain.

In general, if not placed on the ears, Vaccaria seeds could not show any therapeutic function. Therefore, we do not place the seeds on ears instead of the acupuncture points on the body. Because they are small, the seeds cannot give efficient simulations to the patient. In addition, we do not press the seeds to increase unnecessary simulations. As a result, Vaccaria seeds give the patients psychological therapy only. We expect that Vaccaria seeds constitute a good candidate for the control group. We anticipate that electroacupuncture can alleviate acute abdominal pain efficiently, shorten the period of stay in the ED, reduce return visits, and achieve a medical cost reduction.

### Trial status

Recruitment began on April 17, 2017 and was completed by April 16, 2018.

## Data Availability

Not applicable.

## Abbreviations

BPI: Brief pain inventory
ED: Emergency department
NTD: New Taiwan dollar
VAS: Visual analog scale
